# Interaction with activated monocytes enhances cytokine expression and suppressive activity of human CD4^+^CD45RO^+^CD25^+^CD127^low^ regulatory T cells

**DOI:** 10.1186/1479-5876-10-S3-P3

**Published:** 2012-11-28

**Authors:** Gina J Walter, Hayley G Evans, Bina Menon, Bruce W Kirkham, Andrew P Cope, Frederic Geissmann, Leonie S Taams

**Affiliations:** 1 Centre for Molecular and Cellular Biology of Inflammation, King’s College London, London, UK; 2 Dept. Rheumatology, Guy’s & St Thomas’ NHS Foundation Trust, London, UK; 3 Academic Dept. Rheumatology, King’s College London, London, UK

## Introduction

Despite the high frequency of CD4^+^ T cells with a regulatory phenotype (CD25^+^CD127^low^FoxP3^+^) in the joints of patients with rheumatoid arthritis (RA), inflammation persists. Regulatory T cells (Tregs) can be converted into pro-inflammatory IL-17-producing cells by inflammatory mediators, particularly IL-1β.

## Aim

To investigate whether activated monocytes, which are abundantly present in the rheumatic joint and potent producers of IL-1β, induce pro-inflammatory cytokine expression in Tregs and whether this impairs Treg function.

## Materials and methods

The presence and phenotype of CD4^+^CD45RO^+^CD25^+^CD127^low^ T cells (memory Tregs) and CD14^+^ monocytes in the peripheral blood (PB) and synovial fluid (SF) from patients with RA was investigated by flow cytometry. FACS-sorted memory Tregs from healthy controls were co-cultured with autologous *in vitro*-activated monocytes and anti-CD3 monoclonal antibody. Intracellular cytokine expression, phenotype and function were determined by flow cytometry, ELISA and proliferation assays.

## Results

Patients with RA showed higher frequencies of CD4^+^CD45RO^+^CD25^+^CD127^low^ Tregs and activated CD14^+^ monocytes in SF relative to PB. We demonstrate that activated monocytes induced an increase in the percentage of IL-17^+^, IFNγ^+^ and TNF-α^+^, but also IL-10^+^ Tregs. Blocking and reconstitution experiments revealed that the observed increase in IL-17^+^ and IFNγ^+^ Tregs was driven by monocyte-derived IL-1β, IL-6 and TNF-α and was mediated by both CD14^+^CD16^-^ and CD14^+^CD16^+^ monocyte subsets. Despite enhanced cytokine expression, cells maintained their CD25^+^FoxP3^+^CD39^+^ Treg phenotype and showed enhanced capacity to suppress proliferation and IL-17 production by effector T cells.

## Conclusion

Tregs exposed to a pro-inflammatory environment show increased suppressive activity.

